# It is not “just a fracture”

**DOI:** 10.1093/jbmrpl/ziae022

**Published:** 2024-04-09

**Authors:** Peggy M Cawthon, Matthew Drake

**Affiliations:** Research Institute, California Pacific Medical Center, San Francisco, CA, United States 94143; Department of Epidemiology and Biostatistics, University of California San Francisco, San Francisco, CA 94143, United States; Division of Endocrinology, Diabetes and Metabolism, Mayo Clinic, Rochester, MN 55905, United States; Robert and Arlene Kogod Center on Aging, Mayo Clinic, Rochester, MN 55905, United States

**Keywords:** osteoporosis, epidemiology, mortality, fracture, survival

## Abstract

In their recent population–based study of nearly 100 000 patients from Ontario, Canada, who had suffered a fracture, Vincent et al. characterized postfracture survival rates. Their findings showed that overall survival was decreased particularly following fractures of the hip or vertebrae, and was worse in men and the oldest old. They found that relative survival, particularly after hip fracture, is strikingly poor with less than one-third of men and one-half of women surviving for 5 years following hip fracture, survival which was far less than that seen for patients afflicted by both prostate and breast cancers. Importantly, mortality risk increased immediately after fracture occurrence and was highest within 1 month of fracture. Collectively, their data suggest that a short, critical window may exist immediately following fracture in which application of interventions to improve survival is likely to be of greatest value. Future work is urgently needed to identify the best approaches to employ during this critical time period in order to optimize survival in patients who have suffered a fracture.

In their recent population–based study of nearly 100 000 patients from Ontario, Canada, who had suffered a fracture, Vincent et al.^1^ characterized postfracture survival rates. They found that overall survival was decreased following fracture, particularly following fractures of the hip or vertebrae, with the increased mortality risk starting immediately after fracture occurrence. In line with previous studies,^2^^,^^3^ Vincent reported that the greatest mortality occurred after hip fracture (as compared to other anatomical locations), in patients from the oldest age groups, and in men.

Their study has several impactful findings. Notably, the greatest reduction in survival following fracture occurs within 1 month of the event, with the risk of death generally returning to near baseline levels by the end of the first year following fracture. As the authors rightfully note, this strongly suggests that interventions to improve survival following fracture should be initiated immediately after the fracture event and ideally within 1 month, before the patient is discharged from fracture care. However, unlike care that occurs for many other major disease conditions (eg, cardiovascular, cancer), treatment pathways for improving postfracture survival do not typically begin within this critical window.^4^

The reasons for this absence of intervention are complex. Fractures are frequently multifactorial and often occur—especially in the oldest old—in those with related comorbid conditions.^5^ One approach to improve survival following fracture may be to reduce the risk of future fracture by the provision of the osteoporosis medication zoledronic acid, which has been shown to reduce postfracture mortality.^6^ However, it is not clear that it is the fracture itself that results in reduced survival. Rather, it is possible that in many cases, a fracture serves as a sentinel event in the progression of a different serious comorbid condition—for example, worsening Parkinson disease, Alzheimer disease, or diabetes, all of which have the potential to increase the risk for falls, the most common event preceding fracture occurrence. In such cases, additional interventions to improve survival might also focus on optimization of treatment for nonskeletal diseases. As a sentinel event, the fracture itself might therefore be used to focus new clinical attention on potentially unrecognized worsening of other underlying comorbid condition(s) that contributed to fracture occurrence. Additional research is needed to determine if a more comprehensive approach, as suggested above, would improve survival following fracture.

Furthermore, this study and others have shown that relative survival,^2^^,^^3^^,^^7^ particularly after hip fracture, is strikingly poor. Less than one-third of men and one-half of women survived 5 years following fracture, with survival particularly poor in patients from the oldest age groups (>86 years). As the authors note, such survival outcomes are much worse even in comparison to those seen for patients afflicted by some common types of cancer. For example, in Ontario, 94% of men with prostate cancer and 89% of women with breast cancer survive for 5 years post-diagnosis. Yet, while fear of cancer is common,^8^ many patients who suffer a fracture may dismiss the seriousness of their condition when they experience “just a fracture.” Future research is needed to examine whether the use of relative survival data at a population level (to monitor fracture trends and management) or clinically (to help frame prognosis) would ultimately improve outcomes. While sharing such dire statistics in the clinic or hospital setting may increase patients’ fears, appropriate attention to the seriousness of a hip fracture coupled with implementation of concerted efforts to improve postfracture treatment approaches should ideally serve to balance any additional patient concerns.

In summary, the report by Vincent et al. highlights the dire survival that is seen following hip fracture, particularly in the oldest old and in men. Their data suggest that a short, critical window may exist immediately following fracture in which the application of interventions to improve survival is likely to be of greatest value. Future work is urgently needed to identify the best approaches to employ during this critical time period in order to optimize the survival of patients who have suffered a fracture.

## Author contributions

Peggy M. Cawthon (Conceptualization, Writing—original draft) and Matthew Drake (Conceptualization, Writing—review & editing)

## Funding

None declared.

## Conflicts of interest

None declared.
